# Supplementary material for: Identification of CT Imaging Phenotypes of Colorectal Liver Metastases from Radiomics Signatures—Towards Assessment of Interlesional Tumor Heterogeneity
Source: Cancers (Basel). 2022 Mar 24;14(7):1646. doi: 10.3390/cancers14071646 (PMC8997087; doi:10.3390/cancers14071646)
Supplement: Supplementary file 1 [file cancers-14-01646-s001.zip › cancers-1640818-supplementary.pdf]

Supplementary Materials: Identification of CT Imaging Phenotypes of Colorectal Liver Metastases from Radiomics Signatures—Towards Assessment of Interlesional Tumor Heterogeneity

Hishan Tharmaseelan, Alexander Hertel, Fabian Tollens, Johann Rink, Piotr Woźnicki, Verena Haselmann, Isabelle Ayx, Dominik Nörenberg, Stefan O. Schoenberg and Matthias F. Froelich

Supplementary Material S1: List of R packages used.

Package	Version	Comment
readxl	1.3.1	import of data
ComplexHeatmap	2.6.2	heatmap visualization
pheatmap	1.0.12	heatmap
circlize	0.4.13	heatmap color schemes
Boruta	7.0.0	feature selection
dplyr	1.0.7	data manipulation/import
Caret	6.0-90	find correlation
glmnet	4.1-3	Lasso Regression

Supplementary material S2: PCA results

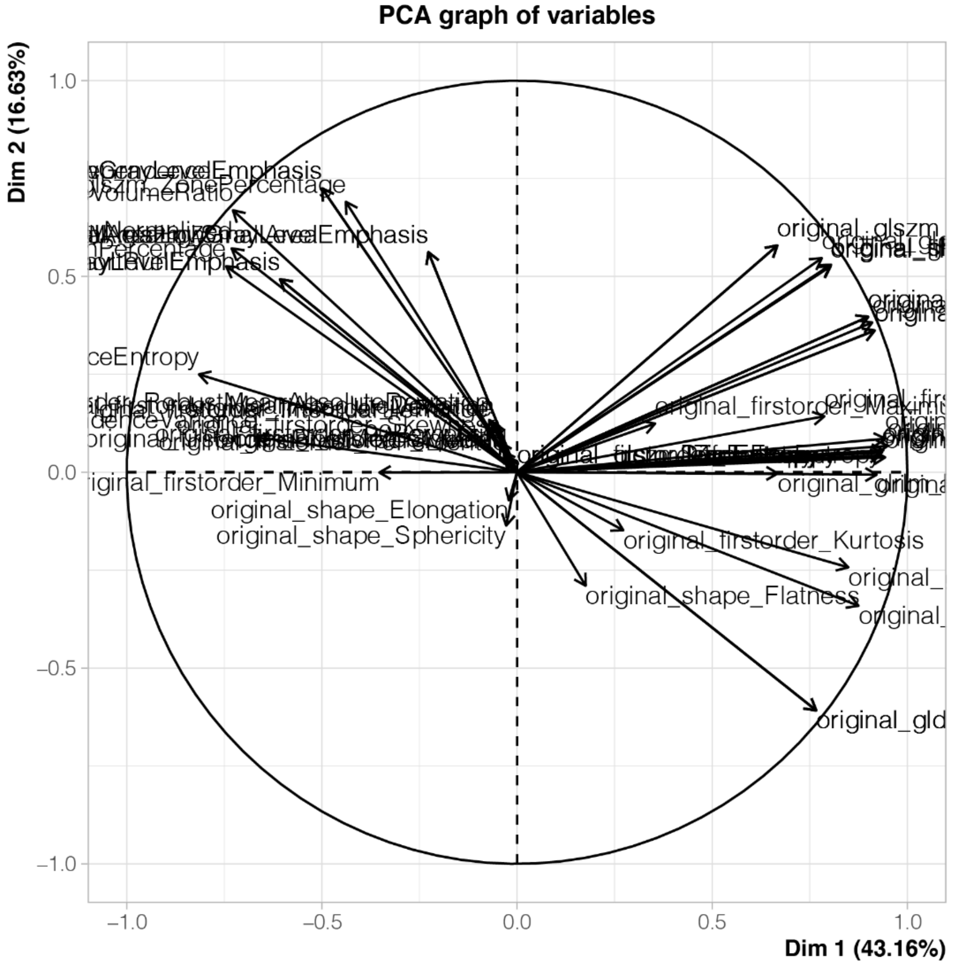

Supplementary material S3: Pyradiomics Settings

Extraction parameters:

{'minimumROIDimensions': 2, 'minimumROISize': None, 'normalize': False, 'normalizeScale': 1, 'removeOutliers': None, 'resampledPixelSpacing': None, 'interpolator': 'sitkBSpline', 'preCrop': False, 'padDistance': 5, 'distances': [1], 'force2D': False, 'force2Ddimension': 0, 'resegmentRange': None, 'label': 1, 'additionalInfo': True}

Enabled filters:

{'Original': {}}

Enabled features:

{'firstorder': [], 'glcm': [], 'gldm': [], 'glrlm': [], 'glszm': [], 'ngtdm': [], 'shape': []}

Supplementary material S4: Results of LASSO regression

(Intercept)	2.60153257
original_shape_Elongation	.
original_shape_Flatness	.
original_shape_Sphericity	.
original_firstorder_Kurtosis	.
original_firstorder_Range	0.08451559
original_firstorder_RootMeanSquared	.
original_firstorder_Skewness	.
original_firstorder_Variance	.
original_glcm_JointEntropy	.
original_gldm_DependenceVariance	-0.21175140
original_glrlm_RunLengthNonUniformity	0.25892993
original_glrlm_ShortRunLowGrayLevelEmphasis	-0.30726364
original_glszm_LargeAreaEmphasis	.
original_glszm_SmallAreaEmphasis	.
